# Current Studies on Molecular Mechanisms of Insulin Resistance

**DOI:** 10.1155/2022/1863429

**Published:** 2022-12-23

**Authors:** Jinli Pei, Baochun Wang, Dayong Wang

**Affiliations:** ^1^Shandong Cancer Hospital and Institute, Shandong First Medical University and Shandong Academy of Medical Sciences, Jinan, Shandong, China; ^2^The First Department of Gastrointestinal Surgery, Hainan General Hospital, Haikou, Hainan 570228, China; ^3^Laboratory of Biopharmaceuticals and Molecular Pharmacology, School of Pharmaceutical Sciences, Hainan University, Hainan 570228, China; ^4^State Key Laboratory of Tropical Biological Resources of the Ministry of Education of China, Hainan University, Hainan 570228, China

## Abstract

Diabetes is a metabolic disease that raises the risk of microvascular and neurological disorders. Insensitivity to insulin is a characteristic of type II diabetes, which accounts for 85-90 percent of all diabetic patients. The fundamental molecular factor of insulin resistance may be impaired cell signal transduction mediated by the insulin receptor (IR). Several cell-signaling proteins, including IR, insulin receptor substrate (IRS), and phosphatidylinositol 3-kinase (PI3K), have been recognized as being important in the impaired insulin signaling pathway since they are associated with a large number of proteins that are strictly regulated and interact with other signaling pathways. Many studies have found a correlation between IR alternative splicing, IRS gene polymorphism, the complicated regulatory function of IRS serine/threonine phosphorylation, and the negative regulatory role of p85 in insulin resistance and diabetes mellitus. This review brings up-to-date knowledge of the roles of signaling proteins in insulin resistance in order to aid in the discovery of prospective targets for insulin resistance treatment.

## 1. Introduction

Diabetes is a complex metabolic disorder associated with increased incidence of cardiovascular and neurological complications. The global prevalence of diabetes has increased rapidly from 4.7% (1980) to 8.7% (2014) [1]. According to the International Diabetes Federation, the number of diabetics worldwide will rise from 451 million in 2017 to 673 million by 2045 [2]. Type II diabetes mellitus (T2D), previously known as non-insulin-dependent diabetes, accounting for 85-90% of the number of diabetic patients, is characterized by impaired insulin sensitivity, diminished beta-cell function, and increased blood glucose levels [3].

One of the key pathogenesis of T2D is insulin resistance, which is also a critical inducement for early prevention and treatment. Insulin resistance is affected by genetic factors including mutations and polymorphism of insulin receptors, insulin receptor substrates, and signal transduction proteins such as PI3K; the binding of which with activated IRS is the critical step linking IR activation to downstream metabolic functions. Extrinsic factors involve circulating metabolites, inflammatory signals, the gut microbiome, and obesity, which are characterized by chronically elevated free fatty acids and may result in lipotoxicity [4–9].

Mutations in the insulin receptor (IR) gene are associated with metabolic syndromes such as the insulin resistance, which can lead to T2D cardiovascular disorders. More than 50 mutations in IR have been identified that are linked to rare forms of insulin resistance [10]. However, T2D caused by the permanent insulin resistance resulted from mutation of IR is uncommon [11]. Insulin resistance is caused primarily by abnormalities in insulin signal transduction.

Insulin signaling downstream of IR is primarily mediated by the insulin receptor substrate (IRS), which activates the phosphatidylinositol 3-kinase (PI3K)-protein kinase B (PKB/AKT) and ERK/MAPK (mitogen-activation protein kinase) pathways, which crosstalk with other signaling pathways [12]. Activation of IR by insulin or insulin-like growth factor (IGF) leads to autophosphorylation of the beta subunit of IR and subsequently phosphorylates IRS at tyrosine residues. Phosphorylated IRS acts as a docking site for proteins with SH2 domains, such as the PI3K regulatory component p85, causing the PI3K-AKT pathway to be activated. Glucose uptake and metabolism as well as fatty acid and protein synthesis are all aided by these intracellular signaling pathways. Binding of IRS with adapter protein Grb2 mediates activation of the ERK pathway which is involved in regulation of genes related to cell survival, cell growth, and differentiation [13]. Ligand-activated IR is internalized and trafficked to early endosome (EE) for dephosphorylation, after which it is degraded or recycled back to the plasma membrane. In insulin resistance and diabetes circumstances, IR trafficking is changed. IR has a spatial preference in triggering its downstream signaling pathway. When internalized, it tends to activate the ERK pathway, while on the plasma membrane, it initiates the PI3K/AKT pathway [14].

Insulin resistance has been linked to alternative splicing abnormalities in IR and IRS gene polymorphism as well as the negatively regulatory effect of p85 in a number of studies.

Extrinsic factors that contribute to insulin resistance have been updated [6]. This review seeks to provide an update on recent research on the molecular mechanisms driving insulin resistance, with a focus on alternative splicing, gene polymorphism, and IRS and PI3K negative regulation.

## 2. Alternative Splicing of IR

**Figure 1:**
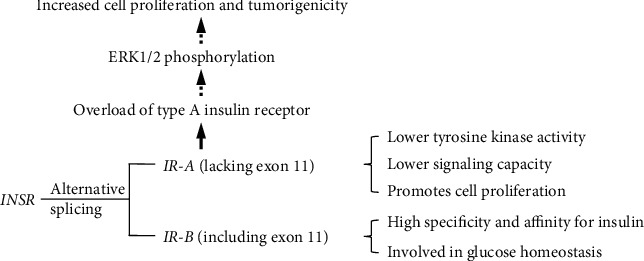
Alternative splicing of *INSR* and aberrant *IR-A*/*IR-B* ratio contributes to insulin resistance.

IR is a transmembrane glycoprotein and a receptor tyrosine kinase that can be activated by insulin and insulin-like growth factor (IGFI and IGFII). IR is encoded by a single gene *INSR*; however, it forms two functionally related but different isoforms, *IR-A* (lacking exon 11) and *IR-B* (including exon 11), due to alternative splicing of exon 11 (Figure 1) [15]. *IR-A* is predominantly expressed in fetal and tumor cells and is involved in cell proliferation, while *IR-B* is found in insulin-sensitive tissue such as pancreatic *β* cells, muscle, kidney, adipose tissue, and liver [16]. There have been reports of functional differences between these two isoforms. IR-B has a high affinity for insulin and is more active in glucose homeostasis, whereas IR-A has a high affinity for IGFII and has reduced tyrosine kinase activity and signaling capacity. IR-A and IR-B activate distinct downstream pathways in pancreatic cells, notably IR-A/PI3K Ia/p70S6 and IR-B/PI3K class II-like/Akt, to regulate insulin transcription and cell survival [17]. Alternative splicing of *INSR* is linked to insulin and glucose levels [18]. The abnormalities in *INSR* splicing and postreceptor signaling are associated with insulin resistance and hyperinsulinaemia [19, 20]. As observed in adipose tissue, *IR-B* is increased in response to weight loss with a strong negative correlation with fasting insulin levels and alternative splicing of *INSR* correlates with the expression of *HNRNPA1*, *SF3A1*, and *SFRS7* [21]. *HNRNPA1* has been previously identified as a known splicing factor to inhibit exon 11 inclusion [22]. Increased expression of CUG-BP, a regulator of pre-mRNA splicing, caused a switch from IR-B to IR-A in skeletal muscle, resulting in reduced insulin signaling activation and contributing to insulin resistance [23].

## 3. Negative Regulation of IR

The level of tyrosine phosphorylation of IR is crucial for controlling insulin signaling, while activation of IR can be inhibited by various proteins for posttranslational modifications, such as tyrosine phosphatase, the Grb protein family (growth-factor-receptor bound protein), SOCS (suppressor of cytokine signaling), and PC-1 or ENPP1 (plasma-cell-membrane glycoprotein-1, also referred to as ectonucleotide pyrophosphatase phosphodiesterase 1). PTP1B (protein tyrosine phosphatase 1B) is the most studied among the tyrosine phosphatases, which interacts with IR and dephosphorylate key tyrosine residues to limit its activity. *PTP1B*gene knockout can increase insulin sensitivity by enhancing IR signaling [24]. Other regulatory proteins, such as SOCS1 and SOCS3, Grb10, Grb14, and PC1, decrease IR binding to IRS or change its kinase activity, thus inhibit IR function [25, 26]. Grb10 or Grb14 overexpression suppresses IRS tyrosine phosphorylation, whereas Grb14 gene knockout improves glucose homeostasis in the liver, white adipose tissues, and heart [27, 28].

SOCS expression has been found to be upregulated in insulin resistance, implying that it plays a role in the feedback control of insulin signaling and the development of diabetes [25]. In the mouse liver, overexpression of SOCS1 reduces insulin sensitivity, whereas suppression of SOCS3 expression improves insulin sensitivity [29]. Furthermore, SOCS directly bind to IR and decrease IRS phosphorylation, reducing insulin signaling, and they further disrupt insulin signaling by promoting ubiquitin-mediated IRS protein degradation [30, 31].

PC-1 expression was found to be higher in T2D patients' muscle, fibroblasts, and adipose tissues, while overexpression of PC-1 in established cell lines inhibited IR autophosphorylation and resulted in insulin resistance [32, 33]. Moreover, PC-1 expression is higher in the fibroblasts isolated from nonobese nondiabetic insulin-resistant subjects, suggesting that PC-1 may play a role in the development of insulin resistance. PC-1 inhibits IR autophosphorylation by directly engaging with 485-599 amino acids (AA) of IR, a tyrosine kinase regulatory domain essential for conformational change, and hence down-regulates subsequent downstream signal transduction, according to further studies [34].

The 121st amino acid of PC-1 is critical for its interaction with IR, and a functional missense nucleotide polymorphism resulting in a lysine to glutamine amino acid change has a stronger interaction with IR and is more effective in lowering IR autophosphorylation [35]. Although the PC-1 gene polymorphism (K121Q) may be a predisposing factor for insulin resistance and T2D, the results have been conflicting, as shown in Table 1. Some studies have made a connection between K121Q and insulin resistance and T2D in the populations such as Ukrainians [36], north Indians [37], South Africans of mixed-ancestry [38], Chinese [39, 40], Americans, Europeans, Africans [41], Asians [42], and Zanjans [43], but others have found no link in Pakistani Punjabis [44], Malaysians [45], Lebanese and Tunisians [46], Chinese [47, 48], and Danish Caucasians [49]. To understand its specific molecular significance in insulin resistance, more research with bigger sample sizes is required.

## 4. IRS

IRS is a crucial mediator of insulin action and serves as a major site for both positive and negative control of insulin signaling transduction. IRS is made up of six members, from IRS1 to IRS6, all of which have relatively similar gene sequences and three-dimensional structures.

The pleckstrin-homology (PH) domain, the adjacent phosphotyrosine-binding (PTB) domain, and the C-terminal domain of them are all extremely similar [51, 52]. The PTB domains bind to the NPEpY sequence of IR, and the C-terminal domain has roughly 20 potential tyrosine phosphorylation sites. On activation of IR, these sites can be phosphorylated and bind to proteins with the Src homology domain 2 (SH2), such as the p85 subunit of the PI3K protein, Grb-2 protein, and the tyrosine protein phosphorylase SHP-2 [53].

As a key node of insulin signaling pathways, the loss of each isoform of IRS leads to varied physiologic results. The distribution and function of the IRS isoforms are summarized in Table 2. *IRS1* gene knockout causes cell differentiation abnormalities in preadipocytes, whereas *IRS2* gene knockout has no effect on cell differentiation but causes nonresponsiveness to insulin-stimulated glucose transport [54]. *IRS1* gene knockout mice exhibit insulin deficiency in muscle tissue, whereas *IRS2* gene knockout animals have insulin deficiency mostly in the liver and generate growth abnormalities in a few tissues, including neurons and pancreatic cells [55, 56]. IRS1 and IRS2 have complimentary effects on activating the AKT signaling pathway but play distinct roles in regulating gene expression, according to IRS1 and IRS2 tissue-specific knockouts studies in the liver.

The downregulation of the IRS1 gene causes the expression of genes involved in gluconeogenesis to increase, whereas the downregulation of the IRS2 gene causes the expression of genes involved in abiogenesis to increase [57]. IRS1 controls glucose uptake, while IRS2 is more closely related to MAPK activation, according to research using small interfering RNAs (siRNAs) to suppress the expression of IRS1 or IRS2 genes in L6 myotubes [58]. Hyperinsulinemia can reduce intracellular levels of IRS1 and IRS2 genes in cell culture models and mouse tissues. The following is the specific mechanism of action: at the transcriptional level, hyperinsulinemia causes IRS1 protein degradation and inhibits IRS2 production.

## 5. Gene Polymorphism of *IRSs*

The prevalent polymorphism of IRS1 is a glycine to arginine substitution in codon 972 (Gly972Arg), which is located between two potential tyrosine phosphorylation sites involved in binding with p85. This polymorphism has been linked to the development of T2D in obese Caucasian children [64], Egyptian patients with chronic hepatitis C virus infection and T2D [65], Kurdish ethnic, and Saudi and Pakistani populations [66–68]. However, no such association with this genetic variant has been found in Sistan and Baluchistan population of Iran [69], Arab, or Berber and Asian Indian populations [70, 71] with T2D (Table 3). The discrepancy could be attributed to differences in racial and ethnic distribution, sample size, and TM subclassifications as well as the inclusion or exclusion of certain insulin resistance confounders.

Gestational diabetes mellitus (GDM), which affects 2-22 percent of all pregnancies, has a genetic background that is similar toT2D, including decreased insulin production and insulin resistance. As a result, similar genetic variations linked to T2D could be applied to predict the risk of GDM. In Saudi [72], Egyptian [73], Iraq [74], and Greek population [75], the association with *IRS1* Gly972Arg GDM has been observed. The Gly1057Asp variation of the IRS2 gene has been widely documented, and it is thought to be linked to insulin resistance, T2D, and obesity. Gly1057Asp inside IRS2 has been linked to greater insulin resistance and T2D in obese Caucasian youngsters, Kurdish ethnics, Iranians, and Asian Indians [66, 76–78], while research in a Turkish women's population suggests that this genetic variation may be linked to GDM as well [79]. However, the genetic variant of IRS2 Gly1057Asp was significantly associated with risk of T2D only in female Bangladeshi population, not males, and the authors were unable to replicate the association of IRS2 Gly1057Asp with obesity [80], while no association with obesity has been found in obese Polish pregnant women [81], implying more appropriate controls for related confounding factors, and a larger sample size is required to confirm this finding.

Six novel IRS4 SNPs have been reported to be linked with BMI in schizophrenic patients [82], while another investigation found no link between IRS4 gene polymorphism and insulin resistance or T2D [83]. The differing results of various researches may be caused by population stratification; adoption of the case-control design would limit the link between allelic polymorphisms in candidate genes and diabetes, which may be related to ethnic or environmental factors produced by population stratification. In addition, the efficacy of different approaches for detecting gene polymorphism varies. To elucidate the significance of gene variation in distinct IRS isoforms in insulin resistance or diabetes mellitus, more research with a larger population and a reasonable design is required.

## 6. Elevated Serine Phosphorylation of IRSs

**Figure 2:**
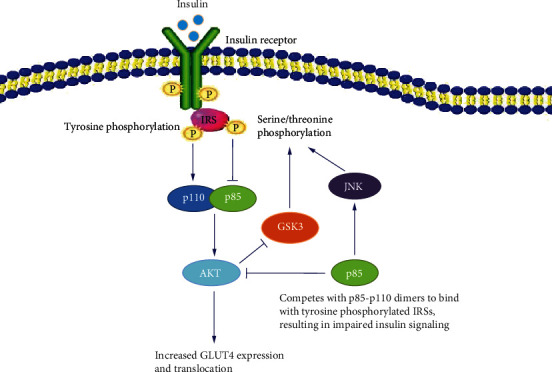
Schematic diagram of potential negative regulation of insulin signal transduction.

There are more than 70 potential serine/threonine phosphorylation sites in IRS protein, in addition to tyrosine phosphorylation sites, which can be induced by a variety of factors, including tumor necrosis factor-*α* (TNF-*α*) [90, 91], c-Jun-amino-terminal kinase (JNK) [92], protein kinase C (PKC) [93], glycogen synthase kinase-3 (GSK-3) [94], SOCS-3 [95], and mitochondrial dysfunction [96]. Serine phosphorylation of IRS1 increases with insulin resistance, and serine hyperphosphorylation of IRS1 is thought to be a negative regulator of insulin signal transduction in general (Figure 2) [97, 98]. Increased serine phosphorylation levels of IR and IRS, as a result of increased circulating fatty acids and ectopic lipid accumulation in muscle and liver, contribute to insulin resistance. Furthermore, elevated levels of circulating fatty acids caused by malnutrition downregulate the insulin signaling pathway by activating serine/threonine phosphorylation kinases including JNK and PKC, as well as impact IRS tyrosine phosphorylation levels by increasing transcription of SOCS proteins [99–101]. IRS1 subcellular localization, trafficking, and degradation as well as interaction with other signaling molecules are all part of the negative regulatory function of serine/threonine phosphorylation. IRS1 phosphorylation at Ser636/639 and Ser307 inhibits IRS1 binding to IR and elevated levels of phosphorylation of serine/threonine in IRS1 reduce IRS1 affinity with the p85 regulatory subunit of PI3K, weakening insulin signal transduction and leading to the symptom of insulin resistance, according to studies [102, 103].

In fact, serine phosphorylation of IRS1 has been shown to have a positive regulatory role, promoting tyrosine phosphorylation while inhibiting serine/threonine phosphorylation at other sites. For example, phosphorylation of IRS1 at Ser629 causes phosphorylation at Ser636 to decrease, adversely regulating IRS1 and increasing insulin activity [104]. While a mutation study found that phosphorylation of *h*Ser1223/*m*1214 (human and rat IRS1) interferes with IRS1's interaction with SHP-2, a negative modulator of IRS1 tyrosine phosphorylation, most likely due to steric hindrance, implying a positive regulatory function [105]. Basal phosphorylation at the serine/threonine of IRS1 potentiates insulin-stimulated tyrosine phosphorylation, whereas hyperphosphorylation inhibits phosphorylation produced by the insulin receptor tyrosine kinase [106, 107].

Serine/threonine phosphorylation at specific sites on IRS provides negative charge, altering protein interactions and reducing downstream cell signaling. By inducing conformational changes in IRS, phosphorylation at certain sites promotes protein interaction and enhances downstream signaling. Furthermore, depending on the time course of insulin action, the interaction status, and the implications of temporal variations in IRS phosphorylation, phosphorylation at specific serine/threonine residues may have varied effects [108]. Phosphorylation of Ser-302/318 is associated with enhanced insulin signaling during the early stages of insulin action, but it is also necessary for attenuating insulin actions during the late stages [109]. Studies into the regulatory effects of serine/threonine phosphorylation patterns on IRS1 activity will aid in understanding insulin resistance.

## 7. PI3K

PI3K inhibitors inhibit practically all of insulin's metabolic activities, including glucose transport, glycogen synthesis, lipid synthesis, and adipocyte differentiation, demonstrating that PI3K plays an important role in insulin signaling [12]. Class I PI3K is a heterodimeric protein consisting of the regulatory subunit p85 and the catalytic subunit p110. It has dual enzymatic activities of both serine/threonine protein kinase and phospholipid kinase. The catalytic subunit p110 is usually in conjunction with the regulatory subunit, and the free p110 is unstable and readily degraded. p85 not only stabilizes p110 but also limits its enzymatic activity by inducing conformational changes [110]. After the regulatory subunit p85 is phosphorylated at Tyr688, the inhibition is abolished. Phosphorylation of Ser608 of p85 by the serine kinase activity of p110 decreases the heterodimer's lipid kinase activity [111]. Additionally, it was discovered in cell culture models that PI3K modulates IRS1 phosphorylation at serine sites and inhibits IRS1 signaling [112].

Insulin sensitivity was increased by a mutation in p85, suggesting that p85 plays a negative function in insulin signal transduction [113, 114]. There are three possible mechanisms which p85 suppresses insulin signaling. The reduction of free monomers in the PI3K-regulatory subunit p85 is the first regulatory mechanism. The amount of p85 is more than p110 and the phosphorylated IRS protein under normal conditions, and there is a balance between the p85-p110 dimer and the free p85 monomers of PI3K. Tyrosine phosphorylation is competed with by free p85. As a consequence of reduced level of free p85, p85-p110 dimers can interact with more phosphorylated IRSs, improving insulin signal transduction [115]. In addition to IRS serine phosphorylation, several investigations have revealed that p85 overexpression is an important molecular mechanism that causes insulin resistance [116]. The isolation of PI3K via the formation of an isolation complex between the p85 monomer and IRS1 to downregulate IR signaling is the second mechanism [117]. Crosstalk between the p85 subunit and the JNK pathway is the third negative regulatory mechanism. Studies have shown that p85 is essential for insulin-stimulated JNK activation. JNK activity is regulated by p85, according to recombination experiments [115]. JNK has been identified in a number of studies to have an important role in metabolism and the development of impaired glucose tolerance and insulin resistance as a result of obesity. Activated JNK phosphorylates the Ser307 residue in IRS1's PTB domain, decreasing its tyrosine phosphorylation and inhibiting the IRS1-PI3K signaling pathway. The phosphorylation level of IRS1 Ser307 induced by JNK was significantly lower in *JNK1* gene knockout mice fed a high fat diet (HFD), suggesting that it may have a protective role in the occurrence of impaired glucose tolerance and insulin resistance [118].

Gene polymorphism of *PI3KR1*, the coding gene of the PI3K p85 subunit, may be associated with GDM [119], T2D [120], insulin resistance, obesity and numerous cancers. A study conducted in Italy found no link between *PI3KR1* and GDM, although the sample size was small, with only 38 pregnant women and 240 controls included [121]. Another study made in China including 334 cases and 367 controls found that *PI3KR1* was involved in abnormal glucose metabolism [119]. A similar finding was found in a Turkish population with 427 diabetic patients and 413 controls, supporting the idea that PI3KR1 is linked to T2D and accompanying symptoms [120].

## 8. Targeting PTP1B in Insulin Resistance Treatment

Tyrosine residues of IR and IRS are dephosphorylated by PTP1B, which is one of the most well-studied tyrosine phosphatases, acting as a crucial negative regulator of the insulin signaling pathways [122]. PTP1B modulation may be valuable as a prospective therapeutic target for the treatment of T2D, and new PTP1B-targeting inhibitors or medications have been developed [123–128]. Due to comparatively lower toxicity, plant-derived medicines have become more prominent. Rampadarath et al. suggested flavonoid C glycosides, particularly orientin, as a possible therapeutic agent in the management of T2D [124]. Low-molecular-weight polymannuronic acid phosphate (LPMP), according to studies by Li et al., may be a promising candidate for an antidiabetic medication since it can reduce oxidative stress and improve insulin sensitivity [127]. The use of accessible synthetic medications, phytochemicals, and potential underlying mechanisms should be highlighted in depth in order to provide management suggestions for insulin resistance in the future [128].

## 9. Conclusion

Since the prevalence of diabetes has increased considerably around the world, understanding the molecular basis of insulin resistance is both theoretically and practically important. More research into the molecular processes of insulin signal transduction, particularly aberrant IR splicing, IRS gene polymorphism, regulatory effects of IRS phosphorylation, and the regulatory function of p85 on insulin activities, is valuable and still needed.

## Figures and Tables

**Table 1:** Association with gene polymorphism of *PC-1* (K121Q) with insulin resistance or T2D in various populations.

Association with gene polymorphism of PC-1 (K121Q)	Population	Reference
Associated with T2D	Ukrainian	[36]
Associated with T2D	South African mixed-ancestry population	[38]
Associated with insulin resistance	North Indian	[37]
Associated with T2D susceptibility	Chinese	[39, 40]
Associated with T2D risk	American, European and African	[41]
Increased susceptibility to diabetic kidney disease	European and Asian	[42]
Enhanced susceptibility to coronary artery disease	South Indian patients with T2D	[50]
Associated with T2D	Zanjan	[43]
No association with T2D	Lebanese and Tunisian	[46]
No association with T2D or obesity	Chinese	[47, 48]
No association with insulin resistance in T2D	Pakistani Punjabi	[44]
No association with T2D	Malaysian	[45]
No association with insulin resistance or T2D	Danish Caucasians	[49]

**Table 2:** Distribution and functions of different IRS isoforms.

Isoforms	Distribution	Activation	Function
IRS1	Widely expressed in various tissues	PI3K, SHP2, and Grb2	Cell differentiation and glucose homeostasis [59]
IRS2	Widely expressed in various tissues	PI3K, SHP2, and Grb2	Cell growth and differentiation, mainly participate in glucose homeostasis in liver [59]
IRS3	Adipocytes and brain	PI3K and SHP2	Cell growth of adipocytes [60]
IRS4	Embryonic tissues	PI3K and Grb2	Cell proliferation and differential and reproductive capacity [61]
IRS5	Mainly in kidney and liver	Undetected	Undefined [62, 63]
IRS6	Skeletal muscle	Undetected	Undefined [63]

**Table 3:** Association with gene polymorphism of *IRS* with insulin resistance or DM in various populations.

Gene polymorphism	Results	Population	Reference
*IRS1*Arg972Gly	Increased insulin resistance	Obese Caucasian children	[64]
*IRS1* Gly972Arg and Ala512Pro	Not associated with T2D	Sistan and Baluchistan population of Iran	[69]
*IRS1* Gly972Arg	Not associate with prediabetes	Northern Vietnamese women	[84]
*IRS1* Gly972Arg	Contributing risk factor for the development of T2D	Egyptian patients with chronic hepatitis C virus infection and T2D	[65]
*IRS1*Gly972Arg	Involved with GDM	Saudi, Iraq, Greek, and Egyptian population	[72–75]
*IRS1*Gly972Arg	Associate with risk of obesity	Obese Polish pregnant women	[81]
*IRS1*Gly972Arg	Associated with T2D	Kurdish ethnic, Saudi, and Pakistani population	[66–68, 85]
*IRS1*Gly972Arg	Correlated with newly diagnosed diabetic patients	Iranian	[86]
*IRS1*Gly972Arg and Ala513Pro	Not associated with T2D	Arab or Berber and Asian Indian populations	[70, 71]
*IRS2*Gly1057Asp	Not associated with T2D	Arab or Berber and Tunisian population	[70, 87]
*IRS2* Gly1057Asp	Associate with T2D only in female	Bangladeshi population	[80]
*IRS2* Gly1057Asp	No association with obesity	Obese Polish pregnant women	[81]
*IRS2*Gly1057Asp	Associated with T2D	Kurdish ethnic	[66]
*IRS2* 1057G/D	Associated with DM	Iranian	[77]
*IRS2*Asp1057Gly	Increased insulin resistance	Obese Caucasian children	[64]
*IRS2*Gly1057Asp	Associated with GDM	Turkish women	[79]
*IRS2*Gly1057Asp	Related with coronary artery disease	Taiwanese	[88]
*IRS2*Gly1057Asp	Increases susceptibility to T2D	Asian Indian	[89]
*IRS4* Val1100Ile, His879Asp, His879Tyr, Ser439Cys, Arg411Glu, and Leu34Phe	Associated with body mass index in patient with schizophrenia	Caucasians	[82]
*IRS4*Leu34Phe, Arg411Gly, Gly584Cys, His879Asp, and Lys883Thr	Not associated with T2D or insulin resistance	Danish Caucasians	[83]
